# Optimum Patient’s Selection for Atrial Fibrillation Ablation Using Echocardiography

**DOI:** 10.3390/diagnostics15212793

**Published:** 2025-11-04

**Authors:** Matteo Cameli, Maria Concetta Pastore, Francesco Morrone, Giulia Elena Mandoli, Giovanni Benfari, Federica Ilardi, Matteo Lisi, Alessandro Malagoli, Simona Sperlongano, Ciro Santoro, Andrea Stefanini, Elena Placuzzi, Annalisa Pasquini, Miriam Durante, Aleksander Dokollari, Michael Y. Henein, Antonello D’Andrea

**Affiliations:** 1 Department of Medical Biotechnologies, Division of Cardiology, University of Siena, Viale Bracci 16, 53100 Siena, Italy; matteo.cameli@unisi.it (M.C.); astefanini94@gmail.com (A.S.);; 2 Section of Cardiology, Department of Medicine, University of Verona, 37134 Verona, Italy; 3 Department of Advanced Biomedical Sciences, Division of Cardiology, Federico II University Hospital, Via S. Pansini 5, 80131 Naples, Italy; 4 Department of Cardiovascular Disease—AUSL Romagna, Division of Cardiology, Ospedale S. Maria delle Croci, Viale Randi 5, 48121 Ravenna, Italy; 5 Division of Cardiology, Nephro-Cardiovascular Department, Baggiovara Hospital, 41126 Modena, Italy; 6 Division of Cardiology, Department of Translational Medical Sciences, University of Campania Luigi Vanvitelli, 80136 Naples, Italy; 7 Department of Advanced Biomedical Sciences, University of Naples Federico II, 80138 Naples, Italy; 8 Department of Cardiovascular and Thoracic Sciences, Fondazione Policlinico Universitario A. Gemelli IRCCS, Università Cattolica del Sacro Cuore, 00168 Rome, Italy; 9 Department of Molecular and Developmental Medicine, University of Siena, 53100 Siena, Italy; 10 Department of Cardiac Surgery Research, Lankenau Institute for Medical Research, Main Line Health, Wynnewood, PA 19096, USA; 11 Department of Public Health and Clinical Medicine, Umeå University, 901 87 Umeå, Sweden; 12 Department of Cardiology, Umberto I Hospital, 84014 Nocera Inferiore, Italy

**Keywords:** atrial fibrillation, catheter ablation, pulmonary vein isolation, echocardiography predictors, recurrence

## Abstract

Catheter ablation (CA) has become a validated technique for treating patients with symptomatic or paroxysmal atrial fibrillation (AF), as recommended by the latest 2024 European society of cardiology (ESC) guidelines, class II level A. The procedure is also recommended for patients with persistent AF without major risk factors for AF recurrence, as an alternative to antiarrhythmic medications class I or III. However, CA carries the risk of AF recurrence in 30–35% of patients, sometimes after the procedure. Multiple factors impact the onset, maintenance, and recurrence of AF after CA, including clinical, biohumoral, echocardiographic, genetic, and lifestyle factors. Beyond traditional predictors, emerging factors such as obstructive sleep apnea syndrome, chronic renal failure, chronic lung disease, physical activity patterns, gut microbiota composition, and epicardial fat thickness significantly influence outcomes. Therefore, optimizing patient’s selection for CA is an important strategy to minimize the risk of AF recurrence. Many echocardiographic parameters emerged as predictors of AF recurrence post-CA, but none stood out as a potential single factor. These factors include traditional markers such as left atrial size by 2D echocardiography, LV ejection fraction, LV diastolic function parameters as well as myocardial deformation addressed by the recently developed speckle tracking analysis. Additionally, the duration and type of AF represent fundamental risk factors, with longstanding persistent AF showing significantly higher recurrence rates compared to paroxysmal forms. Novel biomarkers including MR-proANP, caspase-8, hsa-miR-206, and neurotrophin-3 show promise in enhancing risk prediction capabilities. The aim of this review is to explore the most relevant echocardiographic parameters, including myocardial deformation, that could accurately predict recurrence of AF after CA, while also examining the role of emerging clinical and biochemical predictors in comprehensive patient selection strategies.

## 1. Introduction

Atrial fibrillation (AF) is the most common arrhythmia in the population, with an estimated prevalence of 10% in patients above the age of 80 years [1], which is expected to increase further due to improved clinical and diagnostic tools with beneficial effects on longevity and survival of older patients [2]. Many risk factors contribute to the occurrence of AF, which are either modifiable, such as hypertension, diabetes mellitus, smoking, generous alcohol intake, and excessive physical activity, or are nonmodifiable [3,4], including advanced age, which is a carrier of cardiac fibrotic process [5]. The diagnosis of AF is conventionally made by electrocardiography, but echocardiographic examination of such patients provides more details which could be useful in predicting its potential response to treatments (i.e., medical and/or electrical) as well as its recurrence [6]. Such information is the cornerstone of decision-making for either rhythm control or rate control [6]. The European society of cardiology (ESC) guidelines currently recommended the use of echocardiographic findings to stratify AF patients for either rate or rhythm control, in addition to catheter ablation (CA) procedure. Current guidelines specifically incorporate echocardiographic parameters in patient selection algorithms for AF ablation, with left atrial size and ventricular function being key determinants of procedural candidacy and expected outcomes [7]. CA therapy has been recommended for patients in whom antiarrhythmic medications fail to revert the rhythm back to sinus or fail to control the rate with its related symptoms. It is also recommended as a first line treatment to improve symptoms and quality of life (QoL) in selected patients including, new-onset fast AF, paroxysmal, persistent, or longstanding persistent AF according to the CABANA trial [8]. Despite the high AF ablation procedure success, approximately 35% of patients experience recurrence within 1 year. Several studies attribute such a high recurrence rate to genetic predisposition, obstructive sleep apnea syndrome (OSAS), hypertension, high body mass index (BMI), or reconnection of isolated pulmonary vein segments [9]. It is always the goal of the cardiologist to identify factors that predict the success of the AF ablation procedure and to stratify patients accordingly. Echocardiography may serve as a guide in this context, with an enlarged left atrium being the main predictor of AF relapse after CA. Advanced echocardiographic techniques, i.e., speckle tracking echocardiography (STE), have been proposed as having additional value in the better stratification of these patients. The aim of this review is to provide an overview on the importance of optimizing AF patients’ selection for CA, based on the current available evidence.

## 2. Catheter Ablation

The main objective in the management of patients with AF is to reduce the burden of symptoms and the risk of stroke through rate or rhythm control, respectively [10]. CA was first used in the mid-1980s for patients with supraventricular tachycardia, and only 10 years later it was performed in the first patient with AF [11]. In 1998, the documented CA success rate in AF was 62%, while more recent data show a success rate of 77% [12], which has made CA increasingly used for rhythm management in patients with AF, according to all guidelines [13]. The most used technique for CA remains pulmonary vein isolation (PVI), which has significantly evolved in recent years, e.g., Seitz et al. demonstrated that adding lines and electrocardiographic fraction ablation to conventional pulmonary vein isolation (PVI) increased the success rate at 18 months, with a subsequent reduction in mortality and morbidity [14,15]. Current guidelines place CA as the second line of treatment for patients with symptomatic paroxysmal atrial fibrillation (PAF) (class I recommendations) or persistent AF, particularly those with poorly controlled symptoms, despite receiving optimum antiarrhythmic therapy [9]. Radiofrequency ablation (RFA) in patients with PAF has been found to be similarly effective in aborting AF [16]. CA involves creating a series of point-to-point radiofrequency lesions using cryoenergy or laser, with the catheter been introduced through the femoral vein [17]. The target site of CA is the left atrium, particularly the pulmonary veins and posterior wall, which are the main source of origin of AF [18]. PVI by radiofrequency has been shown to be an effective therapy for treating patients with PAF [19]. However, PVI alone is not sufficient in patients with persistent AF; the posterior wall also plays a role in the maintenance and recurrence of AF, and it shares similar embryological origins with pulmonary veins (PVs). Anatomically, the fibers are oriented in a way that promotes anisotropism, and, thus, re-entry mechanisms. Additionally, late sodium currents and potassium rectifying currents proved to have different characteristics from other sites. This demonstrates how the posterior wall has mechanisms that maintain and promote AF, so it is important to evaluate the degree of fibrosis of the posterior wall for better patient selection [20,21]. The isolation of pulmonary veins may also be achieved using cryoballoons. Three large prospective randomized controlled trials (RCTs) have compared cryoballoon ablation with the Heart Rhythm Society (HRS), the European Heart Rhythm Association (EHRA), and the European Cardiac Arrhythmia Society (ECAS) consensus document on AF ablation, defining procedure success as a period free of symptomatic or asymptomatic AF, atrial tachycardia, or atrial flutter that lasts more than 30 s at 12 months after AF ablation [22]. A meta-analysis has also shown that AF CA in patients without heart failure is associated with a lower rate of hospitalization and recurrence of atrial arrhythmias compared with patients treated with medical therapy. This meta-analysis studied 13 trials with a total of 3856 patients, and a follow-up of 19 months [23]. The early treatment of AF for stroke prevention trial by AF network (EAST-AFNET4), which followed 2789 patients with AF diagnosed within 12 months and with a CHA2DS2-Vasc risk score 2 or more, demonstrated the advantage of early rhythm control (ERC) performed with medication or ablation over rate control [24]. An RCT, with a total of 21,039 participants, showed that CA in the elderly (>75 years) compared with the non-elderly population (<60 years) was associated with higher recurrence rates, probably because of the difference in the left atrial size [25]. A meta-analysis, which studied the impact of age in a similar way, has shown an increased risk related to both the procedure and also all of its complications in the elderly population [26]. In contrast to the thermal CA, pulsed field CA (PFA) creates greater acute tissue changes, without obstructive microvascular damage or intramural hemorrhage, as well as less chronic late gadolinium enhancement (LGE) than thermal CA, which is a marker of myocardial fibrosis. It also results in less complications at the ablation site and better preservation of left atrial (LA) function [27]. Natakani et al. showed a recurrence rate of about 39% in patients undergoing PFA [27]. A meta-analysis of 21 studies with a total of 1559 patients showed that among the risk factors for AF recurrence, there was an increased left atrial volume (LAV) and left atrial volume index (LAVI); therefore, it concluded that echocardiography should be exploited to select patients undergoing CA [28]. In summary, the optimal selection of patients undergoing CA is crucial, to avoid the risks associated with the procedure, including cardiac tamponade, stroke, and pulmonary vein stenosis [29].

### Additional Predictors for AF Recurrence

Beyond traditional echocardiographic parameters, several additional factors significantly influence AF recurrence rates after catheter ablation. Age and gender play crucial roles in determining ablation success rates. Advanced age is associated with increased atrial fibrosis and structural remodeling, while gender-specific differences in atrial anatomy and hormonal influences affect outcomes [30]. The duration and type of AF represent fundamental risk factors for recurrence, with longstanding persistent AF showing significantly higher recurrence rates compared to paroxysmal forms, due to more extensive structural and electrical remodeling [31]. Genetic predisposition influences both AF susceptibility and post-ablation outcomes. Polymorphisms in genes encoding ion channels, structural proteins, and inflammatory mediators have been associated with varying ablation success rates [32]. The choice of ablation technique (radiofrequency vs. cryoballoon vs. pulsed field ablation) and procedural approach significantly impacts long-term outcomes. Complete pulmonary vein isolation and additional substrate modification strategies affect recurrence rates [33]. Obstructive sleep apnea syndrome (OSAS) represents a major modifiable risk factor for AF recurrence, with untreated sleep apnea associated with significantly higher recurrence rates due to intermittent hypoxemia and autonomic dysfunction [34]. Chronic renal failure contributes to recurrence through multiple mechanisms including volume overload, electrolyte imbalances, and accelerated cardiovascular remodeling [35]. Chronic lung disease affects outcomes through hypoxemia, increased pulmonary pressures, and right heart strain [36]. Physical activity demonstrates a complex relationship with AF recurrence—while moderate exercise is beneficial, excessive endurance training may increase recurrence risk through atrial remodeling and autonomic changes [37]. Gut microbiota composition has recently been recognized as a potential predictor of AF recurrence, with specific bacterial profiles associated with inflammatory states and metabolic dysfunction that promote arrhythmia recurrence [38] (Table 1).

## 3. Echocardiography and Atrial Fibrillation

### 3.1. Atrial Fibrillation and Cavity Remodeling

The increase in pressure and volume causes LA stretching with subsequent remodeling. Advanced age, hypertension, and heart failure also cause LA remodeling [53]. Myocardial stretch is associated with inflammation, with its consequences, fibroblasts activation and connective tissue deposition, resulting in contractile dysfunction and further cavity dilatation [54]. Atrial dilatation promotes alteration of ion channels and increases re-entry circuits. Thus, atrial size is an important factor for initiating re-entry arrhythmia, and in supporting maintenance of AF [55,56]. In patients with chronic AF, it has been shown that PV CA can restore sinus rhythm but the success rate is lower than in patients with paroxysmal AF. The procedure success is based on the accurate measurement of the anteroposterior diameter of the LA, before CA, from the parasternal long axis window (55 ± 5 mm vs. <40 mm) [46]. Major studies on the echocardiographic predictors of AF relapse are resumed in Table 2. The 2012 European expert consensus indicates that the LA diameter over 50–55 mm as the upper limit for successful CA [22]. The meta-analysis by Bajraktari et al. demonstrated a lower sinus rhythm maintenance rate in patients with LA diameter > 50 mm, volume > 150 mL and strain less than 19% [57]. However, LA antero—posterior diameter should not always be considered a reliable reflection of true LA size because cavity dilatation may be asymmetric [58]. Hwang et al. showed that, after CA, the LA presents continuous structural and functional changes. These occur mainly in patients with persistent AF (PeAF), having highlighted that pre-CA LAVI is a better predictor of successful outcome, particularly if obtained using 3D echocardiography [47]. A LAVI > 34 mL/m^2^ has proved to have a sensitivity of 70% and a specificity of 91% for predicting AF recurrence after RFCA [48]. Currently LAVI has been recommended as an important predictor of the occurrence and maintenance of AF, having been recognized by ESC guidelines.

**Figure 1:**
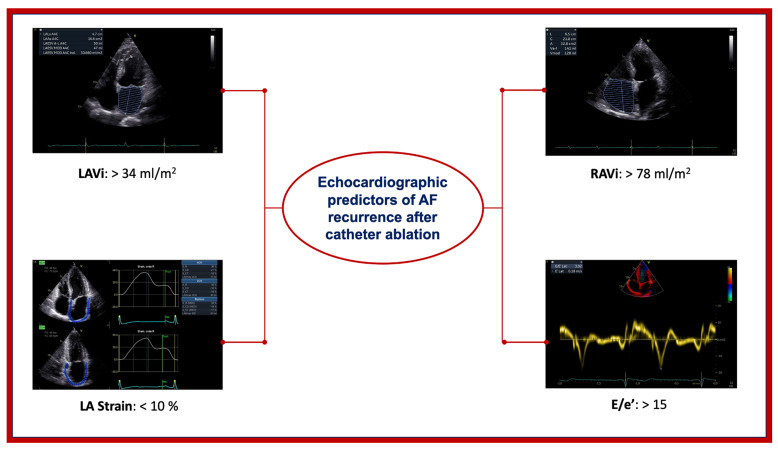
Echocardiographic predictors of atrial fibrillation recurrence after catheter ablation. E/E’, early diastolic wave by transmitral pulsed wave Doppler/average E wave velocity by tissue Doppler imaging in the three points of mitral valve descent; LA, left atrial; LAVI, left atrial volume index; RAVI, right atrial volume index.

In recent years, advanced echocardiographic techniques, such as speckle tracking echocardiography (STE) as a measure of myocardial deformation, have allowed for the objective identification of abnormalities in cardiac function in the early and subclinical stages of various heart diseases [64] (Figure 1).

Several studies have demonstrated that a reduction in LA function, assessed by STE, predicts the presence of myocardial fibrosis, outperforming standard echocardiographic measures such as left atrial ejection fraction (LAEF), transmitral flow, and tissue Doppler imaging (TDI) analysis [65]. The most widely used index is peak atrial longitudinal strain (PALS), a marker of atrial reservoir function, for which the normal cut-off value is 42.2 ± 6.1% (although it varies depending on age and sex) [66,67]. Atrial fibrosis reduces PALS values, thus making it a better marker than the LAV, LA emptying fraction, and E/e’ ratio for predicting the presence of myocardial fibrosis (MF) [49]. Also, identifying atrial regions extensively modified by fibrotic tissue can improve the selection of patients undergoing CA. A very reduced PALS is consistent with severe MF that can reduce the probability of CA success [62]. Fifty-five subjects with AF relapse have been shown to have a significantly reduced LA strain (9.7 ± 2.4% vs. 16.2 ± 3.0%, *p* < 0.001), and the cut-off value of 10% defined the highest risk of recurrence, indicating that PALS was the strongest predictor of AF recurrence [63]. In addition to PALS, an RCT enrolling 118 patients with PAF and PeAF showed that higher strain rate (SR) values were associated with a higher likelihood of maintaining sinus rhythm; this was less likely in patients with PeAF who had lower SR values and post-CA recurrence [59]. A study by Mirza et al. found that a reduction in the LA lateral wall SR at baseline is a sensitive marker of atrial alteration compared with atrial dilatation in patients who had AF recurrence post-CA, particularly those with PAF [61]. A meta-analysis has demonstrated the higher accuracy of SR in predicting AF recurrence post-CA in patients with PAF. The LA strain (range 18.8–25.2%) was strongly associated with AF recurrence; this shows how the degree of strain is related to the degree of fibrosis and that, as evidenced by other studies, the degree of fibrosis is associated with a reduced rate of maintenance of sinus rhythm [68,69]. The LA strain has been shown to be superior to conventional LA measurements in predicting AF recurrence post-CA, with values < 10% strongly associated with recurrence in patients with PeAF. By contrast, LA strain > 14.5% has a high positive predictive value (PPV) in determining the non-recurrence of atrial arrhythmias after two CA procedures [56]. Reant et al. [60] showed that strain and SR imaging can be used in patients with PAF to predict post-CA recurrences, while Schneider et al. [59] used the LA strain and SR immediately after CA, having demonstrated that high values are better associated with the maintenance of sinus rhythm. A three-dimensional global LA strain has been shown to be superior to two-dimensional LA strain as a predictor of post-CA recurrence.

LA function is defined in multiple directions (unlike PALS which is only longitudinal) [70]. In addition to LA dilatation and myocardial deformation parameters, a reduction in atrial conduction velocity can give rise to re-entry circuits, and, hence, promotes AF persistence. Total atrial conduction time (PA-TDI) is a fast and reproducible measure to estimate atrial conduction time as well as mechanical dyssynchrony, which indicates the presence of structural and electrical remodeling [71]. A recent study showed that PA-TDI duration is an independent predictor of AF recurrence after radiofrequency catheter ablation (RFCA) [50]. The right atrium also plays a role in the onset and maintenance of AF. In patients with PAF and concomitant LA dilatation, a right atrial diameter (RAD) < 35.5 mm correlates with a lower rate of post-CA-arrhythmia recurrence [72]. As studied by Moon, increased RA volume index (RAVI) correlates with a higher rate of post-RFCA recurrence; specifically, a RAVI > 78 mL/m^2^ has a sensitivity of 74% and a specificity 68% in predicting post-CA recurrence [73]. Details on the methodology and clinical significance of the echocardiographic parameters to predict AF relapse are given in Table 3.

### 3.2. Epicardial Fat Thickness as a Risk Factor

Epicardial fat thickness (EFT) has emerged as a separate and important echocardiographic risk factor for AF recurrence after catheter ablation [74]. Epicardial adipose tissue represents a metabolically active depot that directly surrounds the heart and coronary vessels. Increased EFT is associated with local inflammatory processes, oxidative stress, and the release of pro-arrhythmic cytokines that promote atrial fibrosis and electrical remodeling [74]. Studies have demonstrated that patients with increased EFT (typically >7–8 mm) show significantly higher rates of AF recurrence following ablation procedures [75]. The mechanism involves direct infiltration of inflammatory mediators from epicardial fat into adjacent myocardial tissue, promoting structural changes that facilitate arrhythmia persistence. EFT can be readily measured using transthoracic echocardiography and provides additional prognostic information beyond traditional parameters like left atrial size and function [52].

### 3.3. AF Ablation in Patients with Left Heart Failure

The efficacy of CA in patients with HFrEF is currently under debate. An RCT comparing patients with systolic dysfunction, isolated diastolic dysfunction, and patients with preserved systolic function has shown a shorter disease-free period in patients with systolic dysfunction (62%) than in those with preserved systolic function. In the latter group, the efficacy of the procedure was superior, if not just comparable to anti-arrhythmic medications, with low procedural risk and improved QoL [76]. In patients with diastolic dysfunction, at 1 year after CA, the results were comparable to those with preserved systolic function but with less improvement in echocardiographic indices [77]. CASTLEAF studied 363 patients and demonstrated that in patients with AF and heart failure, CA was associated with a lower rate of hospitalization and death compared with medical therapy. Moreover, after a 5-year follow-up, 63% of patients undergoing CA remained in sinus rhythm [78]. Diastolic dysfunction plays a significant role in the development of AF and post-CA recurrences, as per the guidelines. LV diastolic dysfunction is assessed using mitral inflow pulsed wave (PW) Doppler, LV TDI Doppler, and LA dimensions [79]. High E/e’, reflecting raised LA pressure, may predict the presence of an arrhythmic substrate located outside the pulmonary veins; this could partially explain AF recurrence [71]. Multiple studies state that the E/e’ ratio is an important parameter in patients with AF, who are undergoing CA. Patients with E/e’ > 15 have been shown to be at higher risk of recurrence than those with E/e’ values < 15. Furthermore, low E/e’ values are likely to be a better indicator for successful CA procedures [51,80].

### 3.4. Atrial Fibrillation and Comorbidity

In addition to structure and function cardiac alterations as predictors of AF recurrence, other factors, including lifestyle, type 2 diabetes, hypertension, obesity, sleep apnea syndrome (OSAS), and advanced age [39], play an important role (Table 1). Consistently high blood pressure (BP) can cause PAF and, if untreated, this may develop into PeAF [39,81]. A Korean study analyzed people who underwent annual cardiovascular screening from 2009 to 2013 with metabolic syndrome, i.e., including not only hypertension but also type 2 diabetes, obesity, and dyslipidemia. In these patients, the number of comorbidities correlated with the risk of the occurrence and recurrence of AF [82]. The mechanisms appear to be multiple, such as increased degree of atrial fibrosis and remodeling, and increased risk of myocardial infarction [83]. Finally, it has been shown that AF frequently occurs in smokers and those with moderate (1–3 drinks/day) or high (>3 drinks/day) alcohol consumption [84].

### 3.5. Atrial Fibrillation and Biohumoral Factors

Clinical and echocardiographic parameters are useful predictors of AF onset or recurrence, but several studies have shown a significant correlation between biomarkers and AF recurrence. The natriuretic peptides (brain natriuretic peptide [BNP] and N-terminal pro-B-type natriuretic peptide (NTproBNP)) are circulating cardiac biomarkers produced in response to conditions of biomechanical stress, negative cardiac remodeling, and fluid overload [85]. A meta-analysis showed how lower NTproBNP values are associated with high rates of sinus rhythm [40]. Kurotobi et al. demonstrated, in 257 patients, that high values of C-reactive protein, a classic biomarker of inflammation, were associated with high rates of recurrence [86]. As previously described, lower PALS values, a novel echocardiography parameter, are associated with a high grade of fibrosis, and, thus, several fibrosis biomarkers have been proposed as predictors of AF recurrence, galectin-3 (Gal-3) and procollagen type III N terminal peptide (PIIINP) being among them. However, future studies are needed to support their role as predictors of AF recurrence after CA [41,67,85].

Recent advances in biomarker research have identified several additional predictors of AF recurrence after catheter ablation [42]. MR-proANP (mid-regional pro-atrial natriuretic peptide) represents a stable fragment of the ANP precursor that provides superior diagnostic and prognostic information compared to traditional natriuretic peptides. Elevated MR-proANP levels reflect atrial wall stress and have been associated with an increased recurrence risk following ablation procedures [87]. Caspase-8 serves as a biomarker of apoptotic activity and cellular turnover. Elevated caspase-8 levels indicate ongoing myocardial cell death and tissue remodeling, processes that promote the arrhythmic substrate responsible for AF recurrence [43]. Hsa-miR-206 belongs to the family of microRNAs that regulate gene expression post-transcriptionally. This specific microRNA is involved in cardiac muscle development and maintenance, with altered expression patterns associated with atrial remodeling, and with increased recurrence risk [88]. Neurotrophin-3 represents a neurotropic factor involved in autonomic nervous system function and cardiac innervation. Altered neurotrophin-3 levels reflect changes in cardiac autonomic balance, which significantly influences AF recurrence patterns following ablation procedures [44]. These emerging biomarkers, when combined with traditional clinical and echocardiographic parameters, may provide enhanced risk stratification for patients undergoing AF ablation procedures [45].

## 4. Conclusions

**Figure 2:**
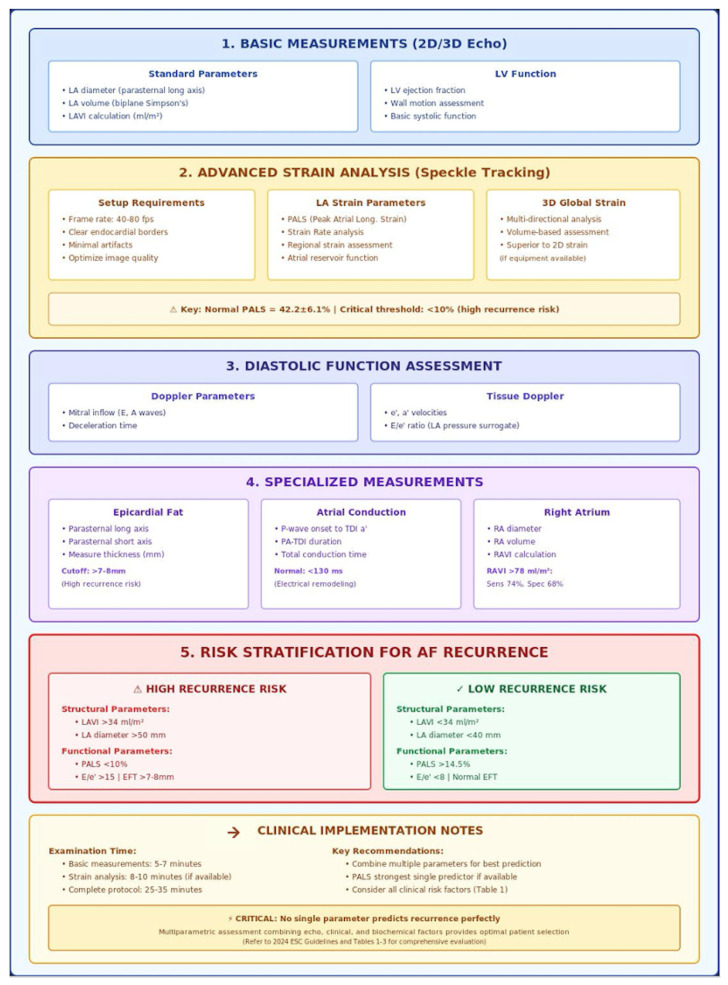
Echocardiographic assessment protocol to evaluate patients with AF after catheter ablation.

Several echocardiographic parameters and biomarkers, as well as comorbidity, play a role in determining the risk of AF recurrence post-CA. Current guidelines already incorporate echocardiographic assessment as a fundamental component of patient selection for AF ablation, recognizing the critical importance of structural and functional cardiac evaluation. Important predictors include not only traditional parameters but also emerging factors such as EFT, duration and type of AF, genetic predisposition, obstructive sleep apnea, chronic renal failure, chronic lung disease, physical activity patterns, and gut microbiota composition. Novel biomarkers, including MR-proANP, caspase-8, hsa-miR-206, and neurotrophin-3, show promise in enhancing risk prediction capabilities beyond conventional markers. However, to identify a single parameter that predicts AF recurrence is still challenging. Therefore, a multiparametric patient evaluation, including conventional measures such as LA size and volume, left ventricular systolic and diastolic function, EFT, and parameters based on STE that reflects LA deformation, are highly recommended to improve patients’ selection (central illustration). Further larger and multicenter studies are warranted to elucidate the role of PALS as a sole parameter guiding CA selection, while also validating the clinical utility of emerging biomarkers and comprehensive risk stratification models that incorporate the full spectrum of clinical, echocardiographic, and biochemical predictors of AF recurrence (Figure 2).

## Figures and Tables

**Table 1:** Predictors of atrial fibrillation recurrence after catheter ablation.

**Clinical Risk Factors**	
Age [31]	
AF duration and type [32]	
Hypertension [39]	
Diabetes mellitus [39]	
Obesity [39]	
Genetic substrate [33]	
Chronic kidney failure [36]	
OSAS [35]	
Chornic lung disease [37]	
Alchol Consumption	
Gut microbiota composition [38]	
**Biohumoral predictors**	
NT-proBNP [40]	
C-reactive protein [41]	
Galectin-3 [42]	
Procollagen type III N terminal peptide [42]	
MR-pro-ANP [43]	
Hsa-miR-206 [44]	
Neurotrophin-3 [45]	
**Echocardiographic predictors of recurrence**	
LA Diameter (antero-posterior) [46]	➢ >50–55 mm
LAVI [47,48]	34 mL/m^2^
PALS [49]	<10%
RAVI [50]	78 mL/m^2^
E/e’ [51]	15
Epicardial fat thickness [52]	**>7–8 mm**

E/e’, early diastolic wave by transmitral pulsed wave Doppler/average E wave velocity by tissue Doppler imaging in the three points of mitral valve descent; Hsa-miR-206, Hsa-microRNA-206; LAVI, left atrial volume index; MR-pro-ANP, mid-regional pro-atrial natriuretic peptide; NT-proBNP, N-terminal pro-brain natriuretic peptide; PALS, peak atrial longitudinal strain; RAVI, right atrial volume index.

**Table 2:** Major studies on echocardiographic parameters for AF recurrence prediction.

Study	Year	Patients	Parameter	Cut-Off	Sensitivity/Specificity	Main Findings
Berruezo et al. [46]	2007	174	LA diameter (AP)	>50 mm	70%/60%	Strongest predictor
Shin et al. [48]	2008	125	LAVI	>34 mL/m^2^	70%/91%	Superior to diameter
Schneider et al. [59]	2008	65	LA SR	-	-	Higher SR → SR maintenance
Reant et al. [60]	2009	40	LA strain	-	-	Predicts PAF recurrence
Mirza et al. [61]	2011	53	LA lateral wall SR	-	-	Sensitive marker
Motoki et al. [62]	2014	55	Global LA strain	10%	-	<10% predicts recurrence
Parwani et al. [63]	2017	118	PALS	10%	82%/76%	Strong predictor in PeAF
Bajraktari et al. [57]	2020	Meta-analysis	LA diameter, LAVI, strain	>50 mm, >150 mL, <19%	-	Combined parameters improve prediction
Wong et al. [50]	2011	387	Epicardial fat thickness	>7 mm	71%/68%	Independent predictor

AP, anteroposterior; LA, left atrial; LAVI, left atrial volume index; PALS, peak atrial longitudinal strain; SR, strain rate.

**Table 3:** Echocardiographic parameters: methodology and clinical significance.

Parameter	Method	Normal	Advantages	Disadvantages	Clinical Utility
LA diameter (AP)	M-mode/2D	<40 mm	Simple, available	Single dimension	Screening
LAVI	Simpson biplane	<34 mL/m^2^	Accurate LA size	Image dependent	Standard
3D LA volume	Real-time 3D	<34 mL/m^2^	No assumptions	Specialized	Superior accuracy
PALS	STE	42.2 ± 6.1%	Early functional change	Angle/vendor dependent	Functional prediction
LA SR	STE	Variable	Dynamic assessment	Complex	Research
Global LA strain	3D STE	-	Multi-directional	Advanced tech	Future standard
Epicardial fat thickness	2D parasternal	<7–8 mm	Simple	Limited views	Metabolic risk
E/e’ ratio	PW/TDI	<8 normal	LV filling pressure	Load dependent	Diastolic function
Total atrial conduction time	TDI	<130 ms	Electrical function	Technical	Conduction assessment

AP, anteroposterior; LA, left atrial; LAVI, left atrial volume index; LV, left ventricular; PALS, peak atrial longitudinal strain; PW, pulsed wave; SR, strain rate; STE, speckle tracking echocardiography; TDI, tissue Doppler imaging.

## Data Availability

No new data were created or analyzed in this study. Data sharing is not applicable to this article.

## References

[1] Escudero-Martínez I., Morales-Caba L., Segura T. (2023). Atrial fibrillation and stroke: A review and new insights. Trends Cardiovasc. Med..

[2] Kornej J., Börschel C.S., Benjamin E.J., Schnabel R.B. (2020). Epidemiology of atrial fibrillation in the 21st century: Novel methods and new insights. Circ. Res..

[3] Kirchhof P., Benussi S., Kotecha D., Ahlsson A., Atar D., Casadei B., Castella M., Diener H.C., Heidbuchel H., Hendriks J. (2016). 2016 ESC Guidelines for the management of atrial fibrillation developed in collaboration with EACTS. Eur. Heart J..

[4] Molina L., Mont L., Marrugat J., Berruezo A., Brugada J., Bruguera J., Rebato C., Elosua R. (2008). Long-term endurance sport practice increases the incidence of lone atrial fibrillation in men: A follow-up study. EP Eur..

[5] Krul S.P., Berger W.R., Smit N.W., van Amersfoorth S.C., Driessen A.H., van Boven W.J., Fiolet J.W., van Ginneken A.C., van der Wal A.C., de Bakker J.M. (2015). Atrial fibrosis and conduction slowing in the left atrial appendage of patients undergoing thoracoscopic surgical pulmonary vein isolation for atrial fibrillation. Circ. Arrhythm. Electrophysiol..

[6] Hindricks G., Potpara T., Dagres N., Arbelo E., Bax J.J., Blomström-Lundqvist C., Boriani G., Castella M., Dan G.A., Dilaveris P.E. (2021). 2020 ESCGuidelines for the diagnosis management of atrial fibrillation developed in collaboration with the European Association for Cardio-Thoracic Surgery (EACTS): The Task Force for the diagnosis management of atrial fibrillation of the European Society of Cardiology (ESC) Developed with the special contribution of the European Heart Rhythm Association (EHRA) of the ESC. Eur. Heart J..

[7] Kuck K.H., Brugada J., Albenque J.P., Van Gelder I.C., Rienstra M., Bunting K.V., Casado-Arroyo R., Caso V., Crijns H.J.G.M., De Potter T.J.R. (2024). 2024 ESC Guidelines for the management of atrial fibrillation developed in collaboration with the European Association for Cardio-Thoracic Surgery (EACTS). Eur. Heart J..

[8] Mark D.B., Anstrom K.J., Sheng S., Piccini J.P., Baloch K.N., Monahan K.H., Daniels M.R., Bahnson T.D., Poole J.E., Rosenberg Y. (2019). Effect of Catheter Ablation vs Medical Therapy on Quality of Life Among Patients with Atrial Fibrillation: The CABANA Randomized Clinical Trial. J. Am. Med. Assoc..

[9] Erhard N., Metzner A., Fink T. (2022). Late arrhythmia recurrence after atrial fibrillation ablation: Incidence, mechanisms and clinical implications. Herzschrittmacherther Elektrophysiol..

[10] Uruthirakumar P., Surenthirakumaran R., Gooden T.E., Lip G.Y.H., Thomas G.N., Moore D.J., Nirantharakumar K., Kumarendran B., Subaschandran K., Kaneshamoorthy S. (2023). The impact of rate and rhythm control strategies on quality of life for patients with atrial fibrillation: A protocol for a systematic review. Syst. Rev..

[11] Buist T.J., Zipes D.P., Elvan A. (2021). Atrial fibrillation ablation strategies and technologies: Past, present, and future. Clin. Res. Cardiol..

[12] Perino A.C., Leef G.C., Cluckey A., Yunus F.N., Askari M., Heidenreich P.A., Narayan S.M., Wang P.J., Turakhia M.P. (2019). Secular trends in success rate of catheter ablation for atrial fibrillation: The SMASH-AF cohort. Am. Hear. J..

[13] Hong K.L., Borges J., Glover B. (2020). Catheter ablation for the management of atrial fibrillation: Current technical perspectives. Open Heart.

[14] Boersma L. (2022). New energy sources and technologies for atrial fibrillation catheter ablation. EP Eur..

[15] Seitz J., Bars C., Théodore G., Beurtheret S., Lellouche N., Bremondy M., Ferracci A., Faure J., Penaranda G., Yamazaki M. (2017). AF Ablation Guided by Spatiotemporal Electrogram Dispersion Without Pulmonary Vein Isolation: A Wholly Patient-Tailored Approach. J. Am. Coll. Cardiol..

[16] Parameswaran R., Al-Kaisey A.M., Kalman J.M. (2021). Catheter ablation for atrial fibrillation: Current indications and evolving technologies. Nat. Rev. Cardiol..

[17] Haegeli L.M. (2012). CardioPulse. Percutaneous radiofrequency catheter ablation of atrial fibrillation. Eur. Heart J..

[18] Quintanilla J.G., Shpun S., Jalife J., Filgueiras-Rama D. (2021). Novel approaches to mechanism-based atrial fibrillation ablation. Cardiovasc. Res..

[19] Dixit S., Marchlinski F.E., Lin D., Callans D.J., Bala R., Riley M.P., Garcia F.C., Hutchinson M.D., Ratcliffe S.J., Cooper J.M. (2012). Randomized ablation strategies for the treatment of persistent atrial fibrillation: RASTA study. Circ. Arrhythm. Electrophysiol..

[20] La Rosa G., Quintanilla J.G., Salgado R., González-Ferrer J.J., Cañadas-Godoy V., Pérez-Villacastín J., Jalife J., Pérez-Castellano N., Filgueiras-Rama D. (2021). Anatomical targets and expected outcomes of catheter-based ablation of atrial fibrillation in 2020. Pacing Clin. Electrophysiol..

[21] Tahir K.S., Mounsey J.P., Hummel J.P. (2018). Posterior Wall Isolation in Atrial Fibrillation Ablation. J. Innov. Card. Rhythm. Manag..

[22] Calkins H., Kuck K.H., Cappato R., Brugada J., Camm A.J., Chen S.A., Crijns H.J., Damiano R.J., Davies D.W., DiMarco J. (2012). 2012 HRS/EHRA/ECAS expert consensus statement on catheter and surgical ablation of atrial fibrillation: Recommendations for patient selection, procedural techniques, patient management and follow-up, definitions, endpoints, and research trial design. Heart Rhythm.

[23] Muhammad Z.K., Safi U.K., Adeel A., Muhammad S.Z., Muhammad U.K., Muhammad S.K., Edo K., Mohamad A. (2020). Meta-Analysis of Catheter Ablation versus Medical Therapy in Patients with Atrial Fibrillation Without Heart Failure. J. Atr. Fibrillation.

[24] Rillig A., Borof K., Breithardt G., Camm A.J., Crijns H.J.G.M., Goette A., Kuck K.H., Metzner A., Vardas P., Vettorazzi E. (2022). Early Rhythm Control in Patients with Atrial Fibrillation and High Comorbidity Burden. Circulation.

[25] Lee W.C., Wu P.J., Chen H.C., Fang H.Y., Liu P.Y., Chen M.C. (2021). Efficacy and Safety of Ablation for Symptomatic Atrial Fibrillation in Elderly Patients: A Meta-Analysis. Front. Cardiovasc. Med..

[26] Kawamura I., Aikawa T., Yokoyama Y., Takagi H., Kuno T. (2022). Catheter ablation for atrial fibrillation in elderly patients: Systematic review and a meta-analysis. Pacing Clin. Electrophysiol..

[27] Nakatani Y., Sridi-Cheniti S., Cheniti G., Ramirez F.D., Goujeau C., André C., Nakashima T., Eggert C., Schneider C., Viswanathan R. (2021). Pulsed field ablation prevents chronic atrial fibrotic changes and restrictive mechanics after catheter ablation for atrial fibrillation. EP Eur..

[28] Shtembari J., Shrestha D.B., Pathak B.D., Dhakal B., Upadhaya Regmi B., Patel N.K., Shantha G.P.S., Kalahasty G., Kaszala K., Koneru J.N. (2023). Efficacy and Safety of Pulsed Field Ablation in Atrial Fibrillation: A Systematic Review. J. Clin. Med..

[29] Njoku A., Kannabhiran M., Arora R., Reddy P., Gopinathannair R., Lakkireddy D., Dominic P. (2018). Left atrial volume predicts atrial fibrillation recurrence after radiofrequency ablation: A meta-analysis. EP Eur..

[30] Guo X., Li J. (2024). Risk and Protective Factors of Recurrence after Catheter Ablation for Atrial Fibrillation. Rev. Cardiovasc. Med..

[31] Haissaguerre M., Jais P., Shah D.C., Takahashi A., Hocini M., Quiniou G., Garrigue S., Le Mouroux A., Le Métayer P., Clémenty J. (1998). Spontaneous initiation of atrial fibrillation by ectopic beats originating in the pulmonary veins. N. Engl. J. Med..

[32] Parvez B., Vaglio J., Rowan S., Muhammad R., Kucera G., Stubblefield T., Carter S., Roden D., Darbar D. (2012). Symptomatic response to antiarrhythmic drug therapy is modulated by a common single nucleotide polymorphism in atrial fibrillation. J. Am. Coll. Cardiol..

[33] Kuck K.H., Brugada J., Furnkranz A., Metzner A., Ouyang F., Chun K.R., Elvan A., Arentz T., Bestehorn K., Pocock S.J. (2016). Cryoballoon or radiofrequency ablation forparoxysmal atrial fibrillation. N. Engl. J. Med..

[34] Kanagala R., Murali N.S., Friedman P.A., Ammash N.M., Gersh B.J., Ballman K.V., Shamsuzzaman A.S.M., Somers V.K. (2003). Obstructive sleep apnea and the recurrence of atrial fibrillation. Circulation.

[35] Naruse Y., Tada H., Satoh M., Yanagihara M., Tsuneoka H., Hirata Y., Ito Y., Kuroki K., Machino T., Yamasaki H. (2013). Concomitant obstructive sleep apnea increases the recurrence of atrial fibrillation following radiofrequency catheter ablation of atrial fibrillation: Clinical impact of continuous positive airway pressure therapy. Heart Rhythm.

[36] Li Y.G., Bisson A., Bodin A., Herbert J., Grammatico-Guillon L., Joung B., Wang Y., Lip G.Y.H., Fauchier L. (2019). C2HEST Score and Prediction of Incident Atrial Fibrillation in Poststroke Patients: A French Nationwide Study. J. Am. Heart Assoc..

[37] Calvo N., Brugada J., Sitges M., Mont L. (2012). Atrial fibrillation and atrial flutter in athletes. Br. J. Sports Med..

[38] Zuo K., Li J., Xu Q., Li K., Hu C., Gao Y., Chen M., Hu R., Liu Y., Chi H. (2019). Dysbiotic gut microbiota and alterations in metabolic patterns are associated with atrial fibrillation. Gigascience.

[39] de Vos C.B., Pisters R., Nieuwlaat R., Prins M.H., Tieleman R.G., Coelen R.J., van den Heijkant A.C., Allessie M.A., Crijns H.J. (2010). Progression from paroxysmal to persistent atrial fibrillation clinical correlates and prognosis. J. Am. Coll. Cardiol..

[40] Xu X., Tang Y. (2017). Relationship between Brain Natriuretic Peptide and Recurrence of Atrial Fibrillation after Successful Electrical Cardioversion: An Updated Meta-Analysis. Braz. J. Cardiovasc. Surg..

[41] Kawamura M., Ito H., Onuki T., Miyoshi F., Watanabe N., Asano T., Tanno K., Kobayashi Y. (2010). Candesartan Decreases Type III Procollagen-N-Peptide Levels and Inflammatory Marker Levels and Maintains Sinus Rhythm in Patients with Atrial Fibrillation. J. Cardiovasc. Pharmacol..

[42] Hijazi Z., Oldgren J., Siegbahn A., Granger C.B., Wallentin L. (2013). Biomarkers in atrial fibrillation: A clinical review. Eur. Heart J..

[43] Gramley F., Lorenzen J., Jedamzik B., Gatter K., Koellensperger E., Munzel T., Pezzella F. (2010). Atrial fibrillation is associated with cardiac hypoxia. Cardiovasc. Pathol..

[44] Ogawa M., Zhou S., Tan A.Y., Song J., Gholmieh G., Fishbein M.C., Luo H., Siegel R.J., Karagueuzian H.S., Chen L.S. (2007). Left stellate ganglion and vagal nerve activity and cardiac arrhythmias in ambulatory dogs with pacing-induced congestive heart failure. J. Am. Coll. Cardiol..

[45] Spronk H.M., De Jong A.M., Verheule S., De Boer H.C., Maass A.H., Lau D.H., Rienstra M., van Hunnik A., Kuiper M., Lumeij S. (2017). Hypercoagulability causes atrial fibrosis and promotes atrial fibrillation. Eur. Heart J..

[46] Berruezo A., Tamborero D., Mont L., Benito B., Tolosana J.M., Sitges M., Vidal B., Arriagada G., Méndez F., Matiello M. (2007). Pre-procedural predictors of atrial fibrillation recurrence after circumferential pulmonary vein ablation. Eur. Heart J..

[47] Hwang J., Park H.S., Han S., Jun S.W., Kang N.Y., Jeon J.H., Choi S.W., Lee C.H., Kim I.C., Cho Y.K. (2020). The impact of catheter ablation of atrial fibrillation on the left atrial volume and function: Study using three-dimensional echocardiography. J. Interv. Card. Electrophysiol..

[48] Shin S.H., Park M.Y., Oh W.J., Hong S.J., Pak H.N., Song W.H., Lim D.S., Kim Y.H., Shim W.J. (2008). Left atrial volume is a predictor of atrial fibrillation recurrence after catheter ablation. J. Am. Soc. Echocardiogr..

[49] Cameli M., Lisi M., Righini F.M., Massoni A., Natali B.M., Focardi M., Tacchini D., Geyer A., Curci V., Di Tommaso C. (2013). Usefulness of atrial deformation analysis to predict left atrial fibrosis and endocardial thickness in patients undergoing mitral valve operations for severe mitral regurgitation secondary to mitral valve prolapse. Am. J. Cardiol..

[50] Wong C.X., Abed H.S., Molaee P., Nelson A.J., Brooks A.G., Sharma G., Leong D.P., Lau D.H., Middeldorp M.E., Roberts-Thomson K.C. (2011). Pericardial fat is associated with atrial fibrillation severity and ablation outcome. J. Am. Coll. Cardiol..

[51] Onishi N., Kaitani K., Amano M., Imamura S., Sakamoto J., Tamaki Y., Enomoto S., Miyake M., Tamura T., Kondo H. (2018). Relationship between left ventricular diastolic dysfunction and very late recurrences after multiple procedures for atrial fibrillation ablation. Heart Vessel..

[52] Mahabadi A.A., Lehmann N., Kalsch H., Robens T., Bauer M., Dykun I., Budde T., Moebus S., Jöckel K.H., Erbel R. (2014). Association of epicardial adipose tissue with progression of coronary artery calcification is more pronounced in the early phase of atherosclerosis: Results from the Heinz Nixdorf recall study. J. Am. Coll. Cardiol. Cardiovasc. Imaging.

[53] Ohtani K., Yutani C., Nagata S., Koretsune Y., Hori M., Kamada T. (1995). High prevalence of atrial fibrosis in patients with dilated cardiomyopathy. J. Am. Coll. Cardiol..

[54] Corradi D., Callegari S., Benussi S., Nascimbene S., Pastori P., Calvi S., Maestri R., Astorri E., Pappone C., Alfieri O. (2004). Regional left atrial interstitial remodeling in patients with chronic atrial fibrillation undergoing mitral-valve surgery. Virchows Arch..

[55] Nattel S., Burstein B., Dobrev D. (2008). Atrial remodeling and atrial fibrillation: Mechanisms and implications. Circ. Arrhythmia Electrophysiol..

[56] van de Vegte Y.J., Siland J.E., Rienstra M., van der Harst P. (2021). Atrial fibrillation and left atrial size and function: A Mendelian randomization study. Sci. Rep..

[57] Bajraktari G., Bytyçi I., Henein M.Y. (2020). Left atrial structure and function predictors of recurrent fibrillation after catheter ablation: A systematic review and meta-analysis. Clin. Physiol. Funct. Imaging.

[58] Faustino A., Providência R., Barra S., Paiva L., Trigo J., Botelho A., Costa M., Gonçalves L. (2014). Which method of left atrium size quantification is the most accurate to recognize thromboembolic risk in patients with non-valvular atrial fibrillation?. Cardiovasc. Ultrasound.

[59] Schneider C., Malisius R., Krause K., Lampe F., Bahlmann E., Boczor S., Antz M., Ernst S., Kuck K.H. (2008). Strain rate imaging for functional quantification of the left atrium: Atrial deformation predicts the maintenance of sinus rhythm after catheter ablation of atrial fibrillation. Eur. Heart J..

[60] Reant P., Lafitte S., Bougteb H., Sacher F., Mignot A., Douard H., Blanc P., Hocini M., Clementy J., Haissaguerre M. (2009). Effect of catheter ablation for isolated paroxysmal atrial fibrillation on longitudinal and circumferential left ventricular systolic function. Am. J. Cardiol..

[61] Mirza M., Caracciolo G., Khan U., Mori N., Saha S.K., Srivathsan K., Altemose G., Scott L., Sengupta P., Jahangir A. (2011). Left atrial reservoir function predicts atrial fibrillation recurrence after catheter ablation: A two-dimensional speckle strain study. J. Interv. Card. Electrophysiol..

[62] Motoki H., Negishi K., Kusunose K., Popović Z.B., Bhargava M., Wazni O.M., Saliba W.I., Chung M.K., Marwick T.H., Klein A.L. (2014). Global left atrial strain in the prediction of sinus rhythm maintenance after catheter ablation for atrial fibrillation. J. Am. Soc. Echocardiogr..

[63] Parwani A.S., Morris D.A., Blaschke F., Huemer M., Pieske B., Haverkamp W., Boldt L.H. (2017). Left atrial strain predicts recurrence of atrial arrhythmias after catheter ablation of persistent atrial fibrillation. Open Heart.

[64] Moharram M.A., Lamberts R.R., Whalley G., Williams M.J.A., Coffey S. (2019). Myocardial tissue characterisation using echocardiographic deformation imaging. Cardiovasc. Ultrasound.

[65] Mandoli G.E., D’Ascenzi F., Vinco G., Benfari G., Ricci F., Focardi M., Cavigli L., Pastore M.C., Sisti N., De Vivo O. (2021). Novel Approaches in Cardiac Imaging for Non-invasive Assessment of Left Heart Myocardial Fibrosis. Front. Cardiovasc. Med..

[66] Cameli M., Miglioranza M.H., Magne J., Mandoli G.E., Benfari G., Ancona R., Sibilio G., Reskovic Luksic V., Dejan D., Griseli L. (2020). Multicentric Atrial Strain COmparison between Two Different Modalities: MASCOT HIT Study. Diagnostics.

[67] Sugimoto T., Robinet S., Dulgheru R., Bernard A., Ilardi F., Contu L., Addetia K., Caballero L., Kacharava G., Athanassopoulos G.D. (2018). Echocardiographic reference ranges for normal left atrial function parameters: Results from the EACVI NORRE study. Eur. Heart J. Cardiovasc. Imaging.

[68] Mondillo S., Cameli M., Caputo M.L., Lisi M., Palmerini E., Padeletti M., Ballo P. (2011). Early detection of left atrial strain abnormalities by speckle-tracking in hypertensive and diabetic patients with normal left atrial size. J. Am. Soc. Echocardiogr..

[69] Ma X.X., Boldt L.H., Zhang Y.L., Zhu M.R., Hu B., Parwani A., Belyavskiy E., Radha Krishnan A.K., Krisper M., Köhncke C. (2016). Clinical Relevance of Left Atrial Strain to Predict Recurrence of Atrial Fibrillation after Catheter Ablation: A Meta-Analysis. Echocardiography.

[70] Liżewska-Springer A., Dąbrowska-Kugacka A., Lewicka E., Drelich Ł., Królak T., Raczak G. (2020). Echocardiographic predictors of atrial fibrillation recurrence after catheter ablation: A literature review. Cardiol. J..

[71] den Uijl D.W., Gawrysiak M., Tops L.F., Trines S.A., Zeppenfeld K., Schalij M.J., Bax J.J., Delgado V. (2011). Prognostic value of total atrial conduction time estimated with tissue Doppler imaging to predict the recurrence of atrial fibrillation after radiofrequency catheter ablation. EP Eur..

[72] Wen S.-, Liu N., Bai R., Tang R.B., Yu R.H., Long D.Y., Sang C.H., Jiang C.X., Li S.N., Wu J.H. (2017). Right atrial diameter and outcome of catheter ablation of atrial fibrillation. J. Interv. Card. Electrophysiol..

[73] Moon J., Hong Y.J., Shim J., Hwang H.J., Kim J.Y., Pak H.N., Lee M.H., Joung B. (2012). Right atrial anatomical remodeling affects early outcomes of nonvalvular atrial fibrillation after radiofrequency ablation. Circ. J..

[74] Batal O., Schoenhagen P., Shao M., Ayyad A.E., Van Wagoner D.R., Halliburton S.S., Tchou P.J., Chung M.K. (2010). Left atrial epicardial adiposity and atrial fibrillation. Circ. Arrhythmia Electrophysiol..

[75] Chao T.F., Suenari K., Chang S.L., Lin Y.-J., Lo L.-W., Hu Y.-F., Tuan T.-C., Tai C.-T., Tsao H.-M., Li C.-H. (2010). Atrial substrate properties and outcome of catheter ablation in patients with paroxysmal atrial fibrillation associated with diabetes mellitus or impaired fasting glucose. Am. J. Cardiol..

[76] Cha Y.M., Wokhlu A., Asirvatham S.J., Shen W.K., Friedman P.A., Munger T.M., Oh J.K., Monahan K.H., Haroldson J.M., Hodge D.O. (2011). Success of ablation for atrial fibrillation in isolated left ventricular diastolic dysfunction: A comparison to systolic dysfunction and normal ventricular function. Circ. Arrhythmia Electrophysiol..

[77] Marrouche N.F., Kheirkhahan M., Brachmann J. (2018). Catheter Ablation for Atrial Fibrillation with Heart Failure. N. Engl. J. Med..

[78] Nagueh S.F., Smiseth O.A., Appleton C.P., Byrd B.F., Dokainish H., Edvardsen T., Flachskampf F.A., Gillebert T.C., Klein A.L., Lancellotti P. (2016). Recommendations for the Evaluation of Left Ventricular Diastolic Function by Echocardiography: An Update from the American Society of Echocardiography and the European Association of Cardiovascular Imaging. J. Am. Soc. Echocardiogr..

[79] Okamatsu H., Ohara T., Kanzaki H., Nakajima I., Miyamoto K., Okamura H., Noda T., Aiba T., Kusano K., Kamakura S. (2015). Impact of left ventricular diastolic dysfunction on outcome of catheter ablation for atrial fibrillation in patients with hypertrophic cardiomyopathy. Circ. J..

[80] O’Keefe E.L., Sturgess J.E., O’Keefe J.H., Gupta S., Lavie C.J. (2021). Prevention and Treatment of Atrial Fibrillation via Risk Factor Modification. Am. J. Cardiol..

[81] Ahn H.J., Han K.D., Choi E.K., Jung J.H., Kwon S., Lee S.R., Oh S., Lip G.Y.H. (2021). Cumulative burden of metabolic syndrome and its components on the risk of atrial fibrillation: A nationwide population-based study. Cardiovasc. Diabetol..

[82] Lee E.Y., Han K., Kim D.H., Park Y.M., Kwon H.S., Yoon K.H., Kim M.K., Lee S.H. (2020). Exposure-weighted scoring for metabolic syndrome and the risk of myocardial infarction and stroke: A nationwide population-based study. Cardiovasc. Diabetol..

[83] Chan Y.H., Chang G.J., Lai Y.J., Chen W.J., Chang S.H., Hung L.M., Kuo C.T., Yeh Y.H. (2019). Atrial fibrillation and its arrhythmogenesis associated with insulin resistance. Cardiovasc. Diabetol..

[84] Larsson S.C., Drca N., Wolk A. (2014). Alcohol consumption and risk of atrial fibrillation: A prospective study and dose-response meta-analysis. J Am. Coll. Cardiol..

[85] Demirel O., Berezin A.E., Mirna M., Boxhammer E., Gharibeh S.X., Hoppe U.C., Lichtenauer M. (2023). Biomarkers of Atrial Fibrillation Recurrence in Patients with Paroxysmal or Persistent Atrial Fibrillation Following External Direct Current Electrical Cardioversion. Biomedicines.

[86] Kurotobi T., Iwakura K., Inoue K., Kimura R., Okamura A., Koyama Y., Toyoshima Y., Ito N., Fujii K. (2010). A pre-existent elevated C-reactive protein is associated with the recurrence of atrial tachyarrhythmias after catheter ablation in patients with atrial fibrillation. EP Eur..

[87] Wachter R., Lahno R., Hamann G.F., Haase B., Weber-Krüger M., Seegers J., Edelmann F., Wohlfahrt J., Gelbrich G., Görlitz A. (2012). Natriuretic peptides for the detection of paroxysmal atrial fibrillation in patients with cerebral ischemia—The Find-AF study. PLoS ONE.

[88] Zhuang J., Wang Y., Tang K., Li X., Peng W., Liang C., Xu Y. (2012). Association between left atrial size and atrial fibrillation recurrence after single circumferential pulmonary vein isolation: A systematic review and meta-analysis of observational studies. Europace.

